# Effects of Dietary Zinc Manipulation on Growth Performance, Zinc Status and Immune Response during *Giardia lamblia* Infection: A Study in CD-1 Mice

**DOI:** 10.3390/nu5093447

**Published:** 2013-09-02

**Authors:** Gemma Iñigo-Figueroa, Rosa O. Méndez-Estrada, Luis Quihui-Cota, Carlos A. Velásquez-Contreras, Adriana Garibay-Escobar, Rafael Canett-Romero, Humberto Astiazarán-García

**Affiliations:** 1 Departamento de Nutrición y Metabolismo, Coordinación de Nutrición, Centro de Investigación en Alimentación y Desarrollo, A.C. Carretera a La Victoria Km 0.6, Hermosillo, Sonora C.P. 83304, Mexico; E-Mails: gemma@estudiantes.ciad.mx (G.I.-F.); romendez@ciad.mx (R.O.M.-E.); 2 Departamento de Nutrición Pública y Salud, Coordinación de Nutrición, Centro de Investigación en Alimentación y Desarrollo, A.C. Carretera a La Victoria Km 0.6, Hermosillo, Sonora C.P. 83304, Mexico; E-Mail: lquihui@ciad.mx; 3 Departamento de Ciencias Químico Biológicas, Universidad de Sonora. Blvd. Luis Encinas y Rosales s/n, Hermosillo, Sonora C.P. 83000, Mexico; E-Mails: velaz@guayacan.uson.mx (C.A.V.-C.); agaribay@guayacan.uson.mx (A.G.-E.); 4 Departamento de Investigación y Posgrado en Alimentos, Universidad de Sonora. Blvd. Luis Encinas y Rosales s/n, Hermosillo, Sonora C.P. 83000, Mexico; E-Mail: rcanett@guayacan.uson.mx

**Keywords:** zinc, *Giardia lamblia*, giardiasis, micronutrients, immune response, IgG, mice

## Abstract

Associations between *Giardia lamblia* infection and low serum concentrations of zinc have been reported in young children. Interestingly, relatively few studies have examined the effects of different dietary zinc levels on the parasite-infected host. The aims of this study were to compare the growth performance and zinc status in response to varying levels of dietary zinc and to measure the antibody-mediated response of mice during *G. lamblia* infection. Male CD-1 mice were fed using 1 of 4 experimental diets: adequate-zinc (ZnA), low-zinc (ZnL), high-zinc (ZnH) and supplemented-zinc (ZnS) diet containing 30, 10, 223 and 1383 mg Zn/kg respectively. After a 10 days feeding period, mice were inoculated orally with 5 × 10^6^ *G. lamblia* trophozoites and were maintained on the assigned diet during the course of infection (30 days). *Giardia*-free mice fed ZnL diets were able to attain normal growth and antibody-mediated response. *Giardia*-infected mice fed ZnL and ZnA diets presented a significant growth retardation compared to non-infected controls. Zinc supplementation avoided this weight loss during *G. lamblia* infection and up-regulated the host’s humoral immune response by improving the production of specific antibodies. Clinical outcomes of zinc supplementation during giardiasis included significant weight gain, higher anti-*G. lamblia* IgG antibodies and improved serum zinc levels despite the ongoing infection. A maximum growth rate and antibody-mediated response were attained in mice fed ZnH diet. No further increases in body weight, zinc status and humoral immune capacity were noted by feeding higher zinc levels (ZnS) than the ZnH diet. These findings probably reflect biological effect of zinc that could be of public health importance in endemic areas of infection.

## 1. Introduction

Nutrition and infection have been linked for centuries and a considerable amount of research has recently been focused on specific nutritional deficiencies [1]. There are links between micronutrient deficiencies and immune impairment [2]. This evidence is strongest for the trace element Zinc (Zn). Zn deficiency could be important for susceptibility to infections, since it is essential for numerous immune functions (Reviewed in [3]). Both epidemiological and clinical experiences indicate an important role of zinc in immunologically mediated host defense [4]. Although nutritional deficiencies are often associated with inadequate food intake and poor dietary quality, many studies have shown that other factors such as intestinal parasites also play an important role as predictors of such deficiencies [5].

A consistent change in level of zinc in the blood of children infected with *G. lamblia* has been noted by some investigators [6,7,8,9,10,11]. A recent study in Peru showed high risk of *Giardia* infection in children aged 2, with 4–8 episodes per year in endemic areas which caused alterations in the absorption of metals, especially Zn [9]. This data has been supported by other authors who have also reported decreased serum Zn levels during giardiasis [6,7,8,9,10,11]. On the other hand, eradication of *G. lamblia* led to a significant improvement in the mean serum Zn levels six months after treatment in schoolchildren from northwestern Mexico [12]. The above-mentioned results show association between giardiasis and zinc levels in human hosts.

This intestinal parasite causes a generally self-limited clinical illness characterized by diarrhea, abdominal cramps, bloating, weight loss, and malabsorption. However, asymptomatic giardiasis with high reinfection rates occurs frequently, especially in developing countries for reasons that remain obscure [13,14]. The study of recurring infectious diseases is a powerful investigative tool; as a rule, the occurrence of a recurrent intestinal infection by *Giardia lamblia*, is a condition that warrants consideration of humoral immune deficiency [15], and a failure to improve may reflect a failure to correct an undefined specific nutrient deficiency, for example, the need for adequate zinc repletion.

Mild to moderate Zn deficiency is now known to occur among children and adults of many countries and is thought to be an important public health problem; its global prevalence was estimated to be 31% in 2004, whereas rates ranged from 4% up to 73% in developing countries [16]. On the other side, giardiasis is endemic in many developing countries, where infection prevalence varies from 20% to 30% [13] and up to 90% of children between the ages 2–4 can become infected at least once.

Moderate zinc deficiency and giardiasis have a strikingly similar geographical distribution and the same people may be experiencing both insults together for a considerable time of their lives. Interestingly, no studies have examined the effects of different dietary zinc levels on the parasite-infected host. Because the immune response elicited to infectious agents normally includes many redundancies, the ultimate consequence of zinc deficiency or supplementation in controlling infection needs to be established in an infected host. Based on all these considerations, the aims of this study were to elucidate whether giardiasis remains a risk factor for zinc deficiency regardless of the level of dietary intake, how this would affect growth performance, and the way the immune system responds to this parasite and shapes the eventual adaptive response according to the dietary zinc level.

## 2. Experimental Section

### 2.1. Mice, Diets and Study Design

**Figure 1:**
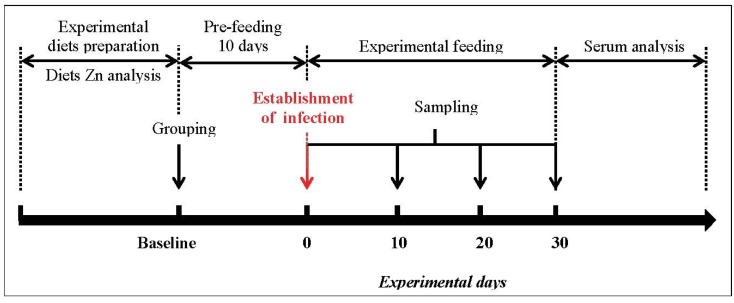
Experimental protocol used in the present study.

An *in vivo* feeding trial was conducted accordingly to the protocol presented in Figure 1 in order to examine the effect of different dietary zinc levels on the growth performance, zinc status and immune response in mice during experimental *Giardia lamblia* infection. Young (6–8 week old) CD-1 male mice were obtained from a colony maintained by the Animal Resource Centre at Universidad de Sonora. Mice were housed in stainless steel cages at a temperature (25 ± 2 °C), humidity (50%–60%) and lightning (lights on from 8:00 to 20:00 h) controlled environment and randomly assigned to either a low-zinc (ZnL, *n* = 20), adequate-zinc (ZnA, *n* = 20), high-zinc (ZnH, *n* = 20) or supplemented-zinc (ZnS, *n* = 20) diet. All diets were prepared based on a modified AIN93G rodent diet [17] with additional zinc as zinc gluconate according to experiment needs (see diet formulations in Table 1).

**Table 1:** Composition of experimental diets.

Ingredient	g/kg diet
Corn starch	653
Casein	200
Corn oil	50
Cellulose	50
Vitamin mix ^1^	10
Mineral mix ^2^	35
Zinc gluconate	*
Choline bitartrate	2

All ingredients purchased from Dyets Inc.; ^1^ AIN-76-A rodent vitamin mix; ^2^ AIN-93-G mineral mix without zinc carbonate; * 0, 0.03, 0.25 and 3.0 g/kg for the ZnL, ZnA, ZnH and ZnS diets respectively.

Mice were fed *ad libitum* the assigned diets for 10 days to accommodate the experimental feeding system. After this adjustment period, half of the mice in each diet group (*n* = 10) were exposed to peroral inoculation of *G. lamblia* trophozoites, while the remaining mice (*n* = 10) were mock infected. The assigned feeding program was followed for the next 30 days post-infection, paying special care with the feed and water to ensure no other sources of infection were introduced to these animals throughout the course of the study.

Body weight was recorded at baseline and then after once a week until the end of the experiment using a precision electronic balance (OHAUS 7124331499). Blood sampling of mice began prior to allocation of dietary treatment and *G. lamblia* infection (B = baseline), and was performed on day 0, 10, 20 and 30 post-infection (Figure 1). Mice were bled from the tail vein and serum was recovered and stored at −20 °C until analysis. The experiments were performed in compliance with the guidelines of the Institutional Animal Care and Use Committee [18].

### 2.2. Establishment of *Giardia lamblia* Infection

*G. lamblia* trophozoites (clone GS/M-83-H7) were obtained from the American Type Culture collection (ATCC 50581). Axenic *G. lamblia* cultures were maintained in TYI-S-33 medium supplemented with 7.0 mL of 10% bovine serum (Bovine Adult SERUM, SIGMA B2771, St. Louis, MO, USA) using a Purifier Class II Biosafety Cabinet (Delta Series, LABCONCO, Kansas City, MO, USA). For experimental inoculation, actively growing trophozoites (48–72 h old cultures) were harvested by being chilled in ice for 20 min. Trophozoites were washed with PBS at pH 7.2 (GIBCO) by 10 min centrifugation (800× *g*) at 4 °C and were resuspended in 500 μL of PBS. Dilutions were prepared with PBS and 0.4% trypan blue (Sigma, Co., St. Louis, MO, USA) to obtain a suspension of 5 × 10^6^ trophozoites in 200 μL. Before inoculation, a 6–9 h fasting period with no water restraint was required to facilitate infection procedure. The trophozoites were inoculated directly into the mice’s duodenum using a syringe fitted with a cannula needle to prevent tissue damage [19].

### 2.3. Diet and Serum Analysis for Zinc Content

All diets were analyzed for zinc content by atomic absorption spectrophotometry (Varian-Spectra AA-20) according to the AOAC 968.08 official method [20]. Before measurement, triplicate samples of each diet were digested with concentrated HNO_3_ (Fisher Scientific, TM grade, Pittsburgh, PA, USA) in a microwave digestor (MDS 2000, CEM Corp., Matthews, NC, USA). The determination of the serum zinc levels was developed according to D’Haese *et al.* [21]. The concentration of the final solutions was measured at 213.9 nm using a hollow cathode zinc lamp. Quality control was monitored using bovine liver standard reference material 1577b (US Department of Commerce, National Institute of Standards and Technology, Gaithersburg, MD, USA) and NIST standard reference material 1577b (US Department of Commerce, National Institute, Gaithersburg, MD, USA). Zinc standards, prepared from a reference solution (Fisher Scientific, Pittsburgh, PA, USA) in 5% nitric acid, were used as internal control. All analyses were conducted in acid-washed glassware.

### 2.4. Immunoglobulin ELISA

To evaluate serum anti-*G. lamblia* IgG production of infected mice, an ELISA was carried out using standard techniques. Briefly, 96 well plates (Corning) were coated overnight with 50 μL (2.5 μg) of soluble *G. lamblia* antigen in 0.1 M sodium bicarbonate buffer pH 9.6. Soluble *G. lamblia* trophozoite antigens were obtained by using the method described by Gottstein *et al*. [22] with slight modifications [23]. Briefly, *G. lamblia* trophozoites from confluent cultures were harvested during log-phase by chilling on ice for 30 min. One hundred million trophozoites were washed three times with sterile phosphate buffer saline (PBS), resuspended in 1.5 mL of PBS, frozen (liquid nitrogen) and thawed (room temperature) three times, and then sonicated (30 cycles for 2 min (Brandon sonifier 250, Shelton, CT, USA) in the presence of protease inhibitor cocktail (23 mM/L 4(2-aminoethyl) benzenesulphonyl fluoride (AEBSF)), 0.3 mM/L pepstatin A, 0.3 mM/L E-64, 2 mM/L bestatin, and 100 mM/L sodium EDTA (Sigma, St. Louis, MO, USA). Cell debris was removed by centrifugation (10,000 *g* for 30 min). The protein concentration of the soluble antigen preparation was determined by the Bradford method (Bio-Rad).

After overnight incubation with soluble *G. lamblia* antigen at 4 °C, plates were washed with PBS-0.05% Tween 20 (PBST), and blocked with PBS-1% BSA for 1 h at room temperature and washed. Mouse serum samples (diluted 1:10 in PBS 1% BSA) from both infected and non-infected mice were added to triplicate wells and incubated for 1 h at room temperature. After washing with PBST, antibody binding was detected with 50 μL of HRP-conjugated goat anti-mouse IgG (1:1000 diluted in PBS 1% BSA) (Sigma, St. Louis, MO, USA). After 1 h of incubation at room temperature, the plates were washed, and developed with 1 mL ABT-S in citrate buffer with 0.03% H_2_O_2_. Optical density was measured at 415 nm with an ELISA reader (Benchmark Microplate Reader, Bio-Rad, Hercules, CA, USA).

### 2.5. Statistical Analysis

All values are given as means ± S.D. Statistical analyses were performed in the statistical software NCSS 2000 (NCSS Statistical Software, Kaysville, UT, USA) either by paired or unpaired Student’s t testing, as appropriate, for analyzing two sets of data and by ANOVA if multiple groups were compared. Differences among means were analyzed using Duncan’s test, with *p* < 0.05 considered as significant.

## 3. Results

### 3.1. Zinc Content of Experimental Diets

All diets were analyzed for zinc content by atomic absorption flame spectrophotometry. The results of the zinc analyses of the four diets indicated the following levels, in mg/kg: 10, 30, 223 and 1383 for the ZnL, ZnA, ZnH and ZnS diets respectively.

### 3.2. Growth Performance as Affected by Dietary Zinc and Infection

Mice in each group were fed with 1 of the 4 experimental diets for 10 days before exposure to *G. lamblia* and during the course of the infection (30 days). The effect of dietary zinc level and infection on body weight is presented on Table 2 where ZnL, ZnA, ZnH and ZnS mice received diets containing 10, 30, 223 and 1383 mg of Zn/kg of diet respectively.

**Table 2:** The effect of dietary zinc and infection on body weight (BW) (g) at day 0 and 30 p.i.

Diet	*Giardia*-free					*Giardia*-infected					
	*n*	Initial BW	Final BW	Gain	*p* *	*n*	Initial BW	Final BW	Gain		*p* *
ZnL	9	34.9 ± 2.1	38.5 ± 1.9 ^b^	3.6	<0.001	10	35.0 ± 2.0	33.9 ± 1.6 ^a^	−1.2		0.214
ZnA	10	35.8 ± 3.0	39.2 ± 2.8 ^b^	3.4	<0.001	10	34.2 ± 2.6	34.5 ± 2.9 ^a^	0.3		0.182
ZnH	10	32.5 ± 1.5	43.6 ± 2.6 ^c^	11.1	<0.001	10	33.0 ± 2.1	42.8 ± 2.6 ^c^	9.6		<0.001
ZnS	8	33.3 ± 1.9	43.1 ± 2.9 ^c^	9.8	0.008	8	34.0 ± 1.7	43.1 ± 2.2 ^c^	9.1		0.002

ZnL = low-zinc, ZnA = adequate-zinc, ZnH = high-zinc, ZnS = supplemented-zinc; Values are expressed as mean ± S.D.; * Paired *t* test, Initial BW *vs*. Final BW, significance at *p* < 0.05; ^a^^,^^b^^,^^c^ Different superscript letters among initial or among final weights indicate significant difference between means, *p* < 0.05.

On the day of the establishment of infection no significant difference was found between the mean weights of the mice from the different groups (*p =* 0.09). At the end of the 30-day period continual weight gain was seen for *Giardia*-free mice in all four dietary groups; in addition, mice fed ZnH and ZnS diets weighed significantly (*p* *<* 0.0001) more than mice fed the ZnL or ZnA ones (43.6 ± 2.3 g and 43.1 ± 2.9 g *vs*. 38.5 ± 1.9 g and 39.2 ± 2.8 g). On the other hand, *Giardia*-infected mice consuming ZnL or ZnA diets essentially failed to grow as demonstrated by the fact that weights remained unchanged (*p* *=* 0.214 and *p* *=* 0.182 for the ZnL and ZnA diets, respectively) after the 30-day period, but there was a significant weight gain in infected mice which received ZnH or ZnS diets during the same period of time (33.0 ± 2.1 g *vs*. 42.8 ± 2.6 g and 34.0 ± 1.7 g *vs*. 43.1 ± 2.2 g for the ZnH and ZnS respectively). This weight improvement was similar to that of the mice not exposed to the infection (9.6 g *vs*. 11.1 g and 9.1 g *vs*. 9.8 g for the ZnH and ZnS infected and non-infected mice respectively).

### 3.3. Serum Zinc Changes Associated with Dietary Zinc Level and Infection

**Figure 2:**
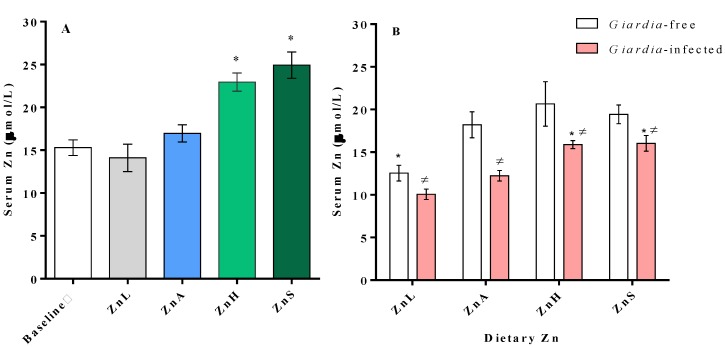
Effects of dietary zinc and infection on serum Zn concentration. Zinc levels in serum of mice following a 10-day feeding period (**A**) and of *Giardia*-free and *Giardia*-infected CD-1 mice following an additional 30-day feeding period p.i. (**B**) with diets containing 10 mg Zn/kg (ZnL), 30 mg Zn/kg (ZnA), 223 mg Zn/kg (ZnH) and 1383 mg Zn/kg (ZnS). Values are expressed as means ± S.D. * Significantly different from ZnA mice (2A) or significantly different from its respective ZnA-non-infected or ZnA-infected control mice (2B), *p* < 0.05. ≠ Significantly different from non-infected dietary control, *p* < 0.05.

Both dietary zinc and infection affected zinc status as assessed by the serum zinc concentration (Figure 2). After 10 day on the feeding regimen, serum zinc concentration was increased in mice fed ZnH and ZnS diets (*p* *<* 0.05), whereas for ZnL and ZnA diets serum zinc level maintained similar to the baseline values (14.1 ± 1.6 µmol/L and 16.8 ± 1.0 µmol/L *vs*. 15.3 ± 0.9 µmol/L, respectively, *p* *=* 0.09) (Figure 2A). Moreover, although a large increase in dietary zinc is observed from the ZnH (223 mg Zn/kg) to the ZnS (1383 mg Zn/kg) diet, serum zinc did not statistically differ between these groups (22.9 ± 0.6 µmol/L and 24.9 ± 1.5 µmol/L, respectively, *p =* 0.13). As expected, infected mice had lower serum zinc levels than non-infected mice in all dietary groups (*p* *=* 0.01) (Figure 2B). However, zinc concentration of *Giardia*-infected mice consuming ZnH or ZnS diets were significantly higher than infected mice fed ZnA diets (15.89 ± 0.46 µmol/L and 16.04 ± 0.92 µmol/L *vs*. 12.24 ± 0.61 µmol/L), and similar to the zinc level of control mice (*Giardia*-free ZnA, 18.21 ± 1.53 µmol/L).

### 3.4. Immune Response as Affected by Dietary Zinc

**Figure 3:**
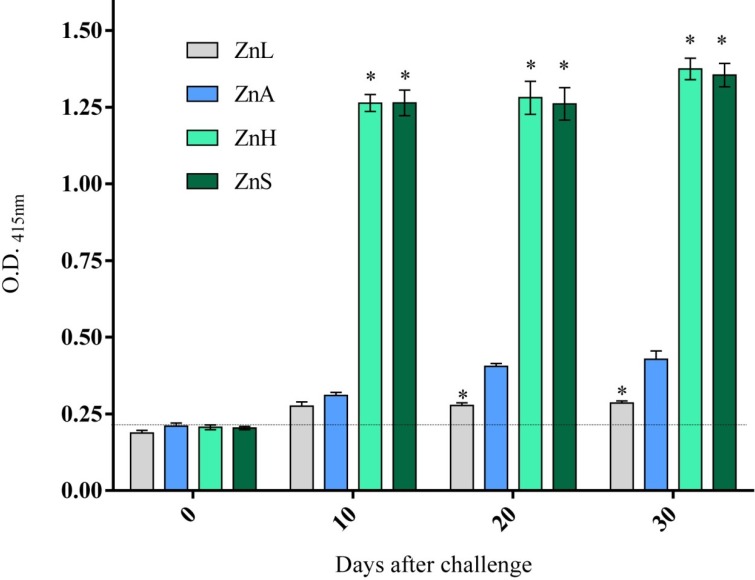
Time-course of parasite-specific IgG antibody response in CD-1 mice after oral immunization with 5 × 10^6^ *G. lamblia* trophozoites. The horizontal dotted line indicates the mean optical density plus two fold standard deviation at 415 nm (O.D. 415 nm) for the negative control and the group of non-infected mice, which represents the cutoff point for positivity of the ELISA. Results are expressed as means ± S.D. from three measurements, each containing pooled sera from 8 to 10 mice. * Significantly different from ZnA mice at each time-point, *p* < 0.05.

To determine the effect of dietary zinc level on immune function, we measured the anti-*G. lamblia* IgG response during the course of primary infection. Antibody response was evaluated at day 0, 10, 20 and 30 p.i. None of the serum samples obtained from uninfected animals had significant levels of antibodies against *G. lamblia* antigens (Data not shown). Figure 3 shows that systemic anti-*G. lamblia* antibody responses became evident at day 10 p.i. in all dietary groups. Nevertheless, ZnH and ZnS mice had notably higher mean levels of *G. lamblia*-specific IgG than ZnA mice (1.263 and 1.281 *vs*. 0.376 optical density units; *p* *=* 0.004). Notably, low zinc diet had little effect on IgG production, since the antibody production of this experimental group was no different from the production of the ZnA group at this time point. However, there was no further increase from day 10 p.i. and IgG levels maintained the same throughout the infection period for this dietary group. On the other hand, IgG values progressively increased throughout the 30-day period for the ZnA, ZnH and ZnS groups, even though differences did not reach statistical significance.

## 4. Discussion

The CD-1 mouse was used as a mode1 of giardiasis to investigate the effects of dietary zinc intake on growth performance, zinc status and immune response during infection. Zinc is a metal with great nutritional importance and is particularly necessary in cell replication and the development of the immune response [3]. Even though zinc deficiencies are characteristically known to initiate anorexia and delay growth in animal models and humans [24,25], in our study body weight gain was not affected by low zinc diet; *Giardia*-free mice fed ZnL or ZnA diets presented a normal growth rate during the experimental 30 day period according to growth data from CD-1 mice suppliers (Charles River Laboratories, San Diego, CA, USA and Harlan Laboratories, Wilmington, MA, USA). On the other hand, mice fed ZnH and ZnS diets manifested rapid growth, with an approximate 30% increase in body weight compared to the 10% increase in mice fed ZnA diet. The explanation for the profound impact of zinc supplemented diets on mouse growth could be attributed to the fact that zinc participates in the regulation of cell proliferation; it is essential for enzyme systems that influence cell division and it also has a role in hormonal regulation of cell division [26]. Dietary zinc supplementation (ZnH and ZnS diets) was possibly effective in improving the body zinc status and the secretory levels/potentiation of growth hormones, thus increasing growth velocity.

Weight loss or reduced weight gain has been associated with giardiasis. In our study weight gain was significantly affected by the *G. lamblia* challenge in mice fed ZnL or ZnA diets, where *Giardia*-free mice had greater weight gain than *Giardia*-infected animals. This is consistent with data from Barthold [27], which reports that although mice infected with *Giardia* are usually asymptomatic, impairment of weight gain is the most common sign of infection. Even though the mice in our study only showed moderate growth retardation, severe weight loss has been reported in lambs infected with *G. lamblia* [28], with similar effects of human giardiasis on body weight [29]. There is increasing evidence to suggest that infection with *Giardia* leads to the development of chronic disorders in the gastrointestinal tract. This impairment of weight gain may be due to nutrient malabsorption, which has been reported in rodent models [19]. The pathophysiological mechanisms that occur during *G. lamblia* infection are not completely understood. Evidence indicates that, shortly after colonization of the small intestinal lumen, *Giardia* trophozoites heighten the rates of enterocyte apoptosis, decrease intestinal barrier function, and these changes lead to diffuse shortening of small intestinal brush border microvilli, maldigestion, and malabsorption, via the activation of CD8+ T-lymphocytes [30].

Animals fed the ZnH and ZnS diets had a higher weight gain than mice fed ZnL and ZnA diets, regardless of whether the animals were infected or not. In this experimental model, dietary zinc level appeared to be more important in regard to growth performance than *Giardia*-infection. Therefore, the growth retardation observed in the ZnL and ZnA *Giardia*-infected groups may have resulted from an interaction of the dietary treatment and the infection, as infection did not significantly affected body weight gain in the ZnH and ZnS groups.

To establish the influence of our diets and *Giardia* infection on zinc status, serum zinc determinations were taken following a 10-day feeding period before the establishment of *Giardia* infection and then 30 day post-infection. The consumption of a ZnL diet for 10 days had no effect on serum zinc level, as mice fed this diet maintained their baseline values, which were similar to that of ZnA mice. Given that mice were at maintenance and that the level of Zn restriction was moderate for a relatively short period, this response is not surprising. However, the consumption of ZnH and ZnS diets resulted in a significant 50%–60% increase in serum zinc levels (Figure 2A). Despite a large increase in dietary zinc is observed from ZnH to ZnS diet, serum zinc did not statistically differ between these groups.

Adjustments in gastrointestinal zinc absorption and intestinal endogenous zinc excretion are the primary means by which the body maintains constant tissue levels of zinc with varying intakes [31]. Zinc transporter systems in enterocytes ZIP4 and ZnT1 [32] respond appropriately to dietary zinc availability and are responsible for a saturable, energy-dependent and regulated uptake of zinc [33]. Studies in experimental rats demonstrate a capacity to maintain a relatively constant content of zinc in the whole body while dietary zinc intakes vary by as much as 10-fold [34]. When the zinc intakes of weanling experimental animals ranged from 10 to 100 mg/kg, the zinc content on the whole remained constant. According to Kirchgessner [34], changes in the concentration of zinc in the whole body present only when very low (<10 mg/kg) or very high (>100 mg/kg) intakes were consumed.

Regarding the influence of *Giardia* infection on zinc status, a consistent change in the level of zinc in the blood of children infected with *G. lamblia* has been reported by some authors [6,7,8,9,10,11]. In our study, *Giardia*-infected mice in all dietary groups had lower serum zinc levels than non-infected mice after 30 days p.i. This is in agreement with other authors whose studies showed that giardiasis may be a risk factor for zinc deficiency in mice regardless of the dietary intake [35]. However, the “extra” zinc provided by the ZnH and ZnS diets helped maintaining the zinc levels near those of the ZnA *Giardia*-free mice. Interestingly, a study by Jendryczko *et al*. [6] shows that in children infested with *G. lamblia* occurs a decrease of zinc absorption in the gastrointestinal tract which causes zinc deficiencies in those children; when compared with non-infected children, mean concentrations of serum zinc carriers, total protein, albumin fraction, transferrin and picolinic acid—a zinc absorption factor in the gastrointestinal tract—were not differing between both studied groups of children. These shows that disturbances might be occurring in the zinc metabolism of infected children and these modifications could be independent of the acute phase response. Therefore, interventions to improve children’s zinc nurture should be considered in populations at risk of zinc deficiency, especially in endemic areas where high reinfection rates of giardiasis are present.

Most infections with *Giardia* are controlled by the host within a few weeks, suggesting the ability of the immune system to control the infection. The high rate of proliferation and differentiation of immune cells requires constant supply of sufficient amounts of zinc. We investigated whether dietary zinc levels adequate for growth would also produce normal response to an infection. Several studies suggest an important role for B cells in clearing *Giardia* infection [36]. B cells differentiate into immunoglobulin secreting plasma cells and hereby induce humoral immunity. For example, *Giardia* infection in humans and mice induce the production of antigiardial antibodies of the immunoglobulin A (IgA), IgM, and IgG isotypes in mucosal secretions and serum, and this specific antibody production correlates with the clearance of infection [37,38,39,40,41]. Such antibodies reach their targets in vivo, since antigiardial IgA and IgG antibodies coat trophozoites in infected mice [37]. It has been shown that zinc deficiency affect B cell lymphopoiesis and also leads to a reduction in antibody-mediated immune defense [42]. However, in our experiment, the consumption of a ZnL diet for a 30 day period had no effect on the capacity of the mice to respond to *G. lamblia* since anti-*G. lamblia* IgG production was comparable to that of the ZnA group. Thus, although these mice consumed a diet with only 30% the amount of zinc in the ZnA diet, it was not sufficient to interfere with their response to *G. lamblia*. This suggests that activated B-cells were able to proliferate and produce antibody in spite of the 30 day period of zinc restriction. These data supports the notion that the Zn deficiency these animals experienced was only moderate, and that the discussed alteration of gastrointestinal physiology giving improved recovery of zinc via transport mechanisms, may have been sufficient to support B-cells proliferation reasonably well in ZnL mice. An extension of the experimental time period might have resulted in a greater loss of body weight and a reduction in immunity in the ZnL mice, as suggested by results of King *et al*. [43], where the thymus of chronic zinc deficient mice were unchanged at day 34, but by day 45 an alteration in lymphopoiesis was observed. Under our experimental conditions, the consumption of a ZnL diet had no quantitative effect on the humoral immune capacity, whereas zinc supplementation led animals to an enhanced immune response, which can be seen through the higher levels of specific IgG antibodies in ZnH and ZnS mice. Our data points in the direction that the zinc concentration used in this experiment seem to have a profound positive effect on humoral immune response. Although zinc is generally regarded as a non-toxic essential metal of particular importance to the immune system, overdosing zinc supplementation can also have a negative impact on immune efficiency [44,45].

Zinc toxicity in rats or mice has not been clearly defined. Studies have indicated that under certain circumstances dietary zinc concentration in excess of 250 mg Zn/kg diet leads to symptoms of toxicity, whereas a more generally recognized toxic concentration is 5000 mg Zn/kg diet [46]. Rather than being a toxic metal ion, zinc is an essential trace element and the human body has efficient mechanisms, to maintain homeostasis over a broad exposure range; consequently, a severe impact on human health by intoxication with zinc is a relatively infrequent event [47].

Results of the present study demonstrate that zinc supplementation can influence the development of adaptive humoral immune response. However, before making recommendations for supplementation, issues of dose and safety need to be addressed. Zinc supplementation with high amounts of Zn (223 and 1383 mg Zn/kg of diet) did not seem to compromise growth performance or specific IgG production in mice. Contrary, there was a higher weight gain and a boosted immune response when compared to diets containing adequate amounts of zinc.

## 5. Conclusions

Taken together, our results showed that *Giardia*-free mice fed a ZnL diet during a 30-day experimental period were able to attain normal growth and antibody-mediated response. *Giardia*-infected mice fed ZnL and ZnA diets present a significant growth retardation compared to non-infected diet controls. Zinc supplementation can avoid this weight loss during *G. lamblia* infection and may be of considerable benefit in improving humoral specific immune response. Clinical outcomes of zinc supplementation during giardiasis include significant weight gain and improved serum Zn levels despite the ongoing of infection. Also, a maximum growth rate and antibody-mediated response were reached in mice fed ZnH diet. No further increases in body weight, zinc status and humoral immune capacity were noted by feeding higher zinc levels (ZnS diet). If the key mechanisms by which zinc exerts its regulatory effect on growth performance and immune response can be identified, successful strategies for preventing/treating this infection may be implemented in the future.
